# Supplementary material for: Insights into the implementation of a whole genome sequencing report form (SRF) to reduce nosocomial SARS-CoV-2 in UK hospitals within an unfolding pandemic: A qualitative process evaluation using normalisation process theory
Source: PLoS One. 2025 Apr 17;20(4):e0321534. doi: 10.1371/journal.pone.0321534 (PMC12005541; doi:10.1371/journal.pone.0321534)
Supplement: Attachment — Submitted filename: Response to reviwers.docx [file pone.0321534.s002.docx]

| **Editor vs reviewers** | **Response** | **Changes** |
| --- | --- | --- |
| Editors comments | | |
| 2. In the ethics statement in the Methods, you have specified that verbal consent was obtained. Please provide additional details regarding how this consent was documented and witnessed, and state whether this was approved by the IRB | Completed | Ethical approval was given by Cambridge South Research Ethics Committee (20/EE/0118) who reviewed the study documentation (participant information sheet, consent form, topic guide and standard operating procedure for data collection).  Verbal consent was attained during the recording prior to data collection beginning by FM asking the participant if they agreed to a series of statements relating to how data were being collected and used for the study.  The date and time consent was obtained were recorded and witnessed by FM and the participant.  Consent for the data to be included in the study was reconfirmed at the end of the interview and an electronic copy of the completed consent form was sent to participants and stored securely by UCL. |
| “This work was supported by funding from the Medical Research Council (MRC) part of UK Research & Innovation (UKRI), the National Institute of Health Research (NIHR) [grant code: MC_PC_19027], and Genome Research Limited, operating as the Wellcome Sanger Institute.”  Please state what role the funders took in the study.  If the funders had no role, please state: "The funders had no role in study design, data collection and analysis, decision to publish, or preparation of the manuscript."  Please include this amended Role of Funder statement in your cover letter; we will change the online submission form on your behalf. | Thank you | The funders had no role in study design, data collection and analysis, decision to publish, or preparation of the manuscript. |
| 4. We note that you have indicated that there are restrictions to data sharing for this study. PLOS only allows data to be available upon request if there are legal or ethical restrictions on sharing data publicly. For more information on unacceptable data access restrictions, please see [http://journals.plos.org/plosone/s/data-availability#loc-unacceptable-data-access-restrictions](https://eur02.safelinks.protection.outlook.com/?url=http%3A%2F%2Fjournals.plos.org%2Fplosone%2Fs%2Fdata-availability%23loc-unacceptable-data-access-restrictions&data=05%7C02%7Cpaul.flowers%40strath.ac.uk%7C99659cc6849342eee6bf08dcdb98d470%7C631e0763153347eba5cd0457bee5944e%7C0%7C0%7C638626698602757304%7CUnknown%7CTWFpbGZsb3d8eyJWIjoiMC4wLjAwMDAiLCJQIjoiV2luMzIiLCJBTiI6Ik1haWwiLCJXVCI6Mn0%3D%7C0%7C%7C%7C&sdata=XViG6YDAuT3bjd8N51vQYikD8Tvj2YBTD3I5wdjPDsY%3D&reserved=0).  Before we proceed with your manuscript, please address the following prompts:  a) If there are ethical or legal restrictions on sharing a de-identified data set, please explain them in detail (e.g., data contain potentially identifying or sensitive patient information, data are owned by a third-party organization, etc.) and who has imposed them (e.g., a Research Ethics Committee or Institutional Review Board, etc.). Please also provide contact information for a data access committee, ethics committee, or other institutional body to which data requests may be sent.  b) If there are no restrictions, please upload the minimal anonymized data set necessary to replicate your study findings to a stable, public repository and provide us with the relevant URLs, DOIs, or accession numbers. For a list of recommended repositories, please see  [https://journals.plos.org/plosone/s/recommended-repositories](https://eur02.safelinks.protection.outlook.com/?url=https%3A%2F%2Fjournals.plos.org%2Fplosone%2Fs%2Frecommended-repositories&data=05%7C02%7Cpaul.flowers%40strath.ac.uk%7C99659cc6849342eee6bf08dcdb98d470%7C631e0763153347eba5cd0457bee5944e%7C0%7C0%7C638626698602770342%7CUnknown%7CTWFpbGZsb3d8eyJWIjoiMC4wLjAwMDAiLCJQIjoiV2luMzIiLCJBTiI6Ik1haWwiLCJXVCI6Mn0%3D%7C0%7C%7C%7C&sdata=0pI3gO42WQyI8kqT3%2BjODqqMC4xotxGqAj0dwrQaVZw%3D&reserved=0). You also have the option of uploading the data as Supporting Information files, but we would recommend depositing data directly to a data repository if possible.  We will update your Data Availability statement on your behalf to reflect the information you provide. | There are ethical restrictions on sharing the de-identified data set in this project.  These relate to the qualitative and sensitive nature of the underlying data and the small number of participants.  Critically, several participants explicitly stated that they did not wish their data to be shared beyond the immediate project (concerned about inquiries into hospital practices).  Further given the small number of participants and project sites, even pseudo-anonymised data may risk identifying participants).  No research ethics committee insisted on this stance but the research participants did. It would be unethical to share data where participants have explicitly requested us not to do so.  Correspondence in this matter can be addressed to  ‘Paul.Flowers@strath.ac.uk’ | Data availability  There are ethical restrictions on sharing data.  The data set is small and rich in qualitative detail. Given the sensitive and confidential nature of the data it cannot be shared with others. Consent to share data beyond the original team was denied by several participants.  Correspondence about this matter can be addressed to ‘Paul.Flowers@strath.ac.uk’ or Professor Ben Jones, Director of research ‘Benedict.jones@strath.ac.uk’ |
| 6. PLOS requires an ORCID iD for the corresponding author in Editorial Manager on papers submitted after December 6th, 2016. Please ensure that you have an ORCID iD and that it is validated in Editorial Manager. To do this, go to ‘Update my Information’ (in the upper left-hand corner of the main menu), and click on the Fetch/Validate link next to the ORCID field. This will take you to the ORCID site and allow you to create a new iD or authenticate a pre-existing iD in Editorial Manager. | Thanks you.  Its hard to sort this out on Editorial manager as when I try to fetch ORCID ID it says:-  An identical, Validated ORCID already exists in the database. An ORCID may be linked to only one record in the database, and so you may have registered another user record in the past and retrieved/validated your ORCID for use with that.  Is it possible to add all authors ORCID IDs? | On the title page we have added the corresponding authors ORCID ID to the manuscript  <https://orcid.org/0000-0001-6239-5616> |
| 7. Your ethics statement should only appear in the Methods section of your manuscript. If your ethics statement is written in any section besides the Methods, please delete it from any other section. | Thank you. We have now deleted our statement on ethics from the ‘declaration section’ |  |
| Reviewer comments | | |
| 1. The use of in-depth interviews and inductive thematic analysis is appropriate for the process evaluation. However, more detailed information on the interview guide and the coding process would enhance the transparency and reproducibility of the study. | Thank you.  We have now added more detail in relation to the nature of the topic guide.  We have also added more detail in relation to the coding process used within the analysis. This is complex but hopefully transparent. | The topic guide was used flexibly in the interviews. Topics covered a range of issues such as how the particular site was responding to COVID at the time of interview, the participants’ role in relation to using the rapid sequencing report, challenges and opportunities associated with the trial, perspectives on the specific nature of the problem the SRF was designed to address, experiences of starting to use the SRF, thoughts on the detail of the SRF, perspectives on how the SRF may have hindered COVID management in the hospital, accounts of how the SRF affected IPC, patient pathways, hospital acquired COVID, accounts of work/life balance, stress and anxiety.  Initial analysis was an iterative process involving both deductive and inductive thematic analysis (18). First, authors PF and FM engaged in multiple data readings and discussions. After this, an initial coding frame was developed containing nine broad categories of data. (1. the perceived problem addressed by the SRF (three sub-codes); 2. details of the context (eleven sub-codes); 3. the acceptability of sequencing for managing infectious disease per se (seven sub-codes); 4. the acceptability of sequencing for managing COVID-19 (seven sub-codes); 5. acceptability of the SRF (seven sub-codes), 6. perspectives of implementing the SRF (seven sub-codes); 7. perspectives on the outcomes and effects of using the SRF (eleven sub-codes); 8. the personal impact of COVID-19 (five sub-codes); and 9. material relating to the trial (seven sub-codes). Some of these nine broad categories were pre-specified (e.g., an a priori deductive focus on the trial for example) others were identified from the data and initial discussions (i.e. the acceptability of sequencing).  Following this initial categorisation of the data, using data from sections five, six, and seven of the coding- frame, inductive themes relating to implementation of the SRF were generated by PF and audited by RL. These included new data-driven themes such as ‘*Passion, will, and infrastructure coalesced around the SRF*’**.** RL and PF then worked collaboratively to map these data-driven themes to NPT constructs and domains (see Table 1) through iterative discussion. Where disagreement occurred, extensive discussion took place to reach a consensus. These inductive themes and their mapping to NPT were then discussed with the wider interdisciplinary trial team. |
| 2. While the authors acknowledge the limitations in generalizability due to the trial context and the pandemic's dynamics, it would be beneficial to discuss potential strategies for scaling up the SRF in more stable conditions. | Thank you  Table 2 provides detailed recommendations about this and we have made changes throughout to emphasise this point. | Table 2, column header and content.  Pg 26 ‘We detailed a series of implications for the future implementation of SRFs (see Table 2) which should be useful for building an evidence base around the use of SRFs within and outside pandemics’. |
| Contextualizing the use of SRF during a viral pandemic: My primary suggestion is that the authors provide greater context by emphasizing that the SRF was used specifically to detect and stop viral transmission during a pandemic. The application of genomic epidemiology for IPC may differ significantly when applied to other pathogens, such as bacteria or fungi, where the transmission dynamics and staff feedback would likely be very different from those reported for this SRF, especially given the unique circumstances of the Alpha variant stage. For example, the timeline of bacterial outbreaks may be protracted in which turnaround time is not as critical as viral outbreaks in which incubation and transmissibility are shorter in duration. The authors could address this distinction in both the introduction and the limitations sections. | Thank you  We have made minor changes to the title, abstract, contributions to the literature, the introduction (page 7), the discussion (page 24), limitations (p28-29) | Contributions to the literature  ‘Very little is known about the use of sequence report forms (SRF) that translate genomic insights concerning the transmission of infectious disease in order to inform infection prevention and control (IPC) responses. Genomic data and SRFs are not routinely used in IPC responses to infections in UK hospitals (either viral, bacterial or fungal). In the context of the COVID-19 pandemic it was possible to evaluate the use of SRFs on IPC for the first time’.  Introduction: (p7) ‘This context, of using an SRF within an unfolding viral pandemic, is important to stress given how different using an SRF may prove to be for either bacterial or fungal infections. For viral infections, for example, turnaround time may be particularly important because incubation and transmission are expedited.’  Limitations (p28-29): ‘Finally, further limitations include the pathogen specificity of the study. We anticipate that using an SRF for bacterial or fungal infections would be different.  Although the challenge of translating genomic insights into IPC responses would be similar, the time sensitivity of the importance of the turnaround time would be lessened given the typically longer incubation periods and slower pace of transmission events typically associated with either bacteria or fungal infections.’ |
| Clarification of SRF's impact Page 16, Line 20: The staff interviewed mentioned “it’s” made a difference. Was the individual specifically referring to the SRF, or could this have been a reference to the enhanced IPC response during the initial waves of COVID-19? For example, it’s possible that the stronger connections formed during the pandemic response, rather than the SRF alone, led to improved outcomes. If the former is the case, perhaps the authors could clarify by adding [SRF] to avoid misinterpretation, as these two interpretations could lead to vastly different understandings of the SRF's impact. | We have checked and the participant was referring to the SRF | ‘*I think it’s [the SRF] enabled the development of collaborations that didn’t previously exist,* |
| Page 18, Line 10: This staff member’s comment, classified under ‘collective action,’ notes that ‘key players are not present,’ but it reads as if they are suggesting that unless frontline healthcare workers are on board, the SRF cannot be successfully implemented. In other words, even if the process as a whole is effective, unless nurses at the bedside change their practices, the IPC interventions cannot lead to sustained changes. This could be clarified. | Thank you. We hope our changes make this clearer now | Pg 20 ‘and a second where key players were not present within these meetings and the interprofessional chain of action across the whole multidisciplinary team (MDT) could not be established and exercised’ |
| Page 19, Line 17: “Understanding the importance of SRF attribution” is crucial, as it directly impacts the perceived effectiveness of interventions, both during the trial and in long-term implementation. If staff believe that the SRF is not driving meaningful change, it’s unlikely that the intervention will be sustained over time. The authors could elaborate on contextual barriers that may have discouraged staff from attributing changes to the SRF. | Thank you. We hope our changes make this clearer now | Pg 22 ‘The overwhelming context of the Alpha variant and the pressures on both the IPC team and the hospital could also have obscured this attribution. In the future ensuring people know that changes to IPC are being driven by the SRF, and that any effective reductions in infections are associated with an SRF will be vital to maintaining its use over time’. |
| Minor Comments:  Page 24, Line 12: Just a comment, but this section is critical for IP&C departments to have takeaways on how to better implement an SRF at their own facility. | Thank you. We hope our minor change amplifies this point. | Pg 27, ‘These insights may prove valuable for others implementing SRFs in their own settings within and outwith future pandemics’. |
